# Treatment of Dens Invagination in a Maxillary Lateral Incisor: A Case Report

**DOI:** 10.7508/iej.2015.03.014

**Published:** 2015-07-01

**Authors:** Azar Heydari, Mona Rahmani

**Affiliations:** a *Endodontic Department, Dental School, Shahid Beheshti University of Medical Sciences, Tehran, Iran*

**Keywords:** Apical Periodontitis, Dens Invagination, Dens Invaginatus, Dens in Dente, Invaginated Teeth, Lateral Incisor, Periapical Lesion

## Abstract

Dens invagination is a developmental anomaly requiring specific treatment approaches. Oehler's* Type III* dens in dente, extends into the root and perforates at the apical area or lateral surface of the root. In this case endodontic treatment of the invaginated tooth was carried out through the central lumen of the invagination with calcium hydroxide without manipulation of the main pulp canal, thereby leaving the tooth vital. The 18-month follow-up examinations were indicative of treatment success; the periapical lesion resolved completely and the tooth remained vital.

**Conclusion:**

Information about the three dimensional anatomy of the teeth especially those with an abnormality is necessary for a successful treatment.

## Introduction

Dens invagination (DI), *aka* dens in dente, *aka* dens invaginatus, is a developmental anomaly resulting from the folding of enamel organ into the dental papilla prior to calcification of dental tissues [1]. The frequency of DI is reported to be 0.04-10% [1]. Its prevalence is the highest in permanent lateral incisors, central incisors, premolars, canines and molars, in a descending order [1]. It more commonly occurs in maxilla than in mandible and in permanent rather than deciduous teeth.[1]

Depth of invagination may vary from a deep cingulum pit to a deep split extending to the apex [1]. DI is categorized into two groups of coronal and radicular types with the coronal type being more prevalent [2, 3]. The coronal form has 3 main types based on the intensity of the defect (classified by Oehler): *type I;* the invagination limited to the crown of the tooth, *type II*; the invagination has extended beyond the cemento-enamel junction (CEJ) and may be connected to the pulp or not, but there is no communication to periradicular tissues and *type III;* the invagination extends into the root and perforates the apical or lateral surface of the root without any communication with the pulp [1-4]. In this type, the enamel coating the invagination is replaced by cementum at the perforation zone. This perforation allows for a direct communication between the oral cavity and the periradicular tissues; thus, it usually causes an inflammatory lesion in spite of a vital pulp [2]. After tooth eruption, the invagination communicates with the oral cavity and the tissues in the lumen become necrotic [2]. In cases that the invaginations extends to the periapical region, the lumen contents or caries must be removed and calcium hydroxide (CH) paste should be placed into the lumen to prevent adjacent pulp involvement [5-7]. Both the invagination and the main pulp should be treated if pulpal involvement has occurred [5, 7, 8]. In open apex cases, apexification with CH or mineral trioxide aggregate (MTA) plug usually has a successful outcome. In cases that are not responsive to conservative endodontic treatment, retrograde periapical surgery is required [5, 8]. Diluted invaginations usually have abnormal crown and must be extracted [4].

This article describes the treatment of a *type III* DI in a maxillary lateral incisor without involving the main pulp canal.

## Case Report

A 16 year-old female was referred to the Department of Endodontics at School of Dentistry, Isfahan University of Medical Sciences, mentioning no remarkable medical history with her chief complaint being swelling, pain and sensitivity to thermal changes in the left side of anterior maxillary segment. 

**Figure 1:**
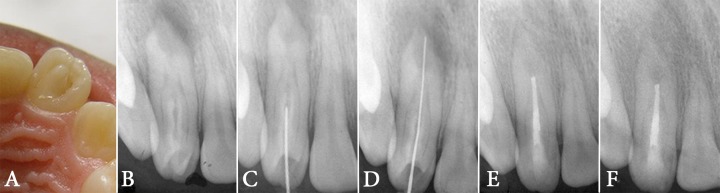
***A) ***The protuberated cingulum in the palatal aspect of the left maxillary lateral incisor, ***B)*** Periapical radiolucency around the apex of the laterla incisor with *type III* dens in dente, ***C) ***Entrance into the lumen without involving the main canal, ***D) ***Working length determination, ***E) ***Obturation of the lumen and ***F) ***Follow-up image after 18 months; the periapical lucency has disappeared

On clinical inspection, the crown of the left maxillary lateral incisor seemed larger than its counterpart on the right side and had a protuberated cingulum (similar to talon cusp) in the palatal aspect. The crown had a labial inclination (Figure 1A). There was a notable localized swelling in the buccal vestibule and the lateral incisor was painful on percussion and palpation. Parallel radiography revealed periapical radiolucency associated with the root of the maxillary left lateral incisor (Figure 1B). An enamel invagination extending beyond the CEJ was noticed in the crown. The enamel invagination had opened into the periodontal ligament in the middle part of the root. Thus, it was categorized as a *type III* invagination based on Oehler’s classification (Figure 1B). Cold test with ice stick and electric pulp tester (Vitality Scanner, SybronEndo, Boston, MA, USA) showed that the tooth was vital and responded to cold stimulation with a sharp transient pain.

Local anesthesia was administered using injection of 2% lidocaine with 1:80000 epinephrine (Darupakhsh, Tehran, Iran) into the buccal vestibule and in palatal mucosa. The tooth was still sensitive to cold indicating the failure of anesthesia. Anesthesia with another infiltration, infra-orbital nerve block and periodontal ligament injections also failed; however the tooth’s response to cold test became milder. After achieving partial anesthesia, preparation of the access cavity started using a diamond fissure bur (Diatech, Heerbrugg, Switzerland). Entry to the central lumen was done through cutting the protuberated cingulum (Figure 1C). Necrotic tissue was detected after entrance into the lumen. Rubber dam was placed and a #15 initial K-file (Mani, Tochigi, Japan) was introduced into the lumen. The working length was measured with an electronic apex locator (Root ZX apex locator, J. Morita USA, Inc., Irvine, CA, USA). The apex locator revealed that the lumen ended in the periodontal ligament in middle root area. The radiographs taken at different angles confirmed the working length (Figure 1D).

Cleaning and shaping of the lumen was done with the master apical file (MAF) set at #55 and #2 and 3 Gates Glidden drills (Mani, Tochigi, Japan) were used in a descending order for coronal flaring. Irrigation was applied using 2.5% NaOCl solution. Creamy paste of CH (Golchai, Tehran, Iran) was placed into the canal and the access cavity was sealed with Coltozol (Ariadent, Asia Chemi Teb. Co., Tehran, Iran). 

One week later, the intracanal CH was replaced with a thick paste and the access cavity was sealed with light cured glass ionomer (Fuji II LC, GC Corporation, Tokyo, Japan). The CH paste was exchanged monthly. The bone radiolucency disappeared after 6 months. In the final appointment, the lumen was irrigated using 2.5% NaOCl and sterile saline. Obturation was done with lateral condensation of gutta-percha cones and AH-26 sealer (Dentsply, Tulsa Dental, and Tulsa, OK, USA). The access cavity was permanently restored with light cured composite resin (Z100, 3M ESPE, Premier, Norristown, PA, USA) and bonding (SingleBond, 3M ESPE, Premier, Norristown, PA, USA) (Figure 1E).

The patient was dismissed and put on a regular follow-up. After 6, 12 and 18 months complete resolution of the periapical radiolucency was detected (Figure 1F). Clinical examination revealed no swelling and no sensitivity to percussion or palpation. The tooth responded to thermal tests but the responses were transient and within the normal range.

## Discussion

This report represented the treatment protocol of a *class III* DI. The teeth with these anomalies usually need a specific treatment plan that may be different from a normal teeth [9]. Radiographic and clinical examination of the present tooth showed that the invagination had extended through a central lumen which was completely separated from the main pulp canal by a layer of integrated dentin. The main pulp canal was like a cylinder with a barrier of dentin that acted as a pathway for the passage of oral bacteria to the periodontium.

The main pulp was vital and inflamed while the central lumen contents were necrotized and infected. Pulpitis might have occurred due to the infection in the central lumen and passage of bacterial byproducts from the infected lumen to the main pulp through the dentinal tubules. Therefore, healing of the main pulp could be expected by removing the contents of the central lumen. Thus, treatment plan included debridement and cleaning of the central lumen while keeping the pulp vital.

A review study by Hulsmann [4] stated that in cases where there is no communication between the invagination lumen and the main canal (like Oehler’s *class III* DI) and if the pulp is

not involved, single treatment of the central lumen is adequate. Some cases with favorable response to such treatments have been reported and discussed by Eldeeb [10], Mangani and Ruddle [11], Khabbaz *et al.* [12] and Keles and Cakici [7]. Sausa* et al.* [13], observed a good response to surgical treatment and retrograde filling of the root end by amalgam after 15 years of follow-up; although, tooth discoloration was obvious. On the other hand, da Silva* et al. *[14] advised concomitant orthograde and retrograde treatments. In the presented case, long-term CH therapy was carried out for the following purposes:

- Achieving a clean lumen without any infectious contents and elimination of bacteria from the dentinal tubules connecting the two spaces (central lumen and the surrounding pulp canal) [15, 16].
- Inducing the formation of a hard tissue barrier at the end of the central lumen in order to prevent the extrusion of obturating materials. (similar to apexification) [17-20].
- Induction of reparative/sclerotic dentinogenesis on dentinal wall between central lumen and peripheral pulp [21, 22].
- To ensure the resolution of pulpitis symptoms, elimination of the need for endodontic treatment of the main pulp canal and make sure than the tooth responds fairly to the selected treatment plan.

Complete resolution of the periradicular lesion, swelling of buccal vestibule and symptoms of pulpitis after 6, 12 and 18 months confirmed the success of treatment. It seems that excessive root enlargement and its extended innervations were responsible for the failure of anesthetic induction. Further investigations are required to assess the innervations of invaginated teeth and their anesthesia.

## Conclusion

Diagnosis and treatment of teeth with abnormalities like dens invaginatus should be done as soon as possible to prevent more complications. Moreover, knowledge about the three dimensional anatomy of the teeth especially in cases of abnormality is necessary for a successful treatment.
